# Control of M. tuberculosis ESAT-6 Secretion and Specific T Cell Recognition by PhoP

**DOI:** 10.1371/journal.ppat.0040033

**Published:** 2008-02-15

**Authors:** Wafa Frigui, Daria Bottai, Laleh Majlessi, Marc Monot, Emmanuelle Josselin, Priscille Brodin, Thierry Garnier, Brigitte Gicquel, Carlos Martin, Claude Leclerc, Stewart T Cole, Roland Brosch

**Affiliations:** 1 UP de Pathogénomique Mycobactérienne Intégrée, Institut Pasteur, Paris, France; 2 Unité de Génétique Moléculaire Bactérienne, Institut Pasteur, Paris, France; 3 Dipartimento di Patologia Sperimentale, Biotecnologie Mediche, Infettivologia ed Epidemiologia, University of Pisa, Pisa, Italy; 4 Unité de Régulation Immunitaire et Vaccinologie, Institut Pasteur, Paris, France, Institut National de la Santé et de la Recherche Médicale, Unité 883, Paris, France; 5 Unité de Génétique Mycobactérienne, Institut Pasteur, Paris, France; 6 Grupo de Genetica de Micobacterias, Departamento de Microbiologica, Facultad de Medicina, Universidad de Zaragoza, Spain; 7 CIBER Enfermedades Respiratorias, Spain; 8 Global Health Institute, Ecole Polytechnique Fédérale de Lausanne, Lausanne, Switzerland; University of Washington, United States of America

## Abstract

Analysis of mycobacterial strains that have lost their ability to cause disease is a powerful approach to identify yet unknown virulence determinants and pathways involved in tuberculosis pathogenesis. Two of the most widely used attenuated strains in the history of tuberculosis research are Mycobacterium bovis BCG (BCG) and Mycobacterium tuberculosis H37Ra (H37Ra), which both lost their virulence during in vitro serial passage. Whereas the attenuation of BCG is due mainly to loss of the ESAT-6 secretion system, ESX-1, the reason why H37Ra is attenuated remained unknown. However, here we show that a point mutation (S219L) in the predicted DNA binding region of the regulator PhoP is involved in the attenuation of H37Ra via a mechanism that impacts on the secretion of the major T cell antigen ESAT-6. Only H37Ra “knock-ins” that carried an integrated cosmid with the wild-type *phoP* gene from M. tuberculosis H37Rv showed changes in colony morphology, increased virulence, ESAT-6 secretion, and induction of specific T cell responses, whereas other H37Ra constructs did not. This finding established a link between the PhoP regulator and ESAT-6 secretion that opens exciting new perspectives for elucidating virulence regulation in M. tuberculosis.

## Author Summary

Mycobacterium tuberculosis, the causative agent of human tuberculosis is an extremely successful human pathogen in spite of its lack of classical virulence factors, such as toxins. The pathogenesis of this bacterium is closely linked with its ability to circumvent destruction by the host cell, which depends on a large variety of mycobacterial lipids and secreted proteins. Genome comparison of fully virulent strains with closely related, but attenuated strains that have lost their ability to cause disease is a powerful approach to identify factors contributing to mycobacterial virulence. In this study we have compared the virulent paradigm strain of tuberculosis research M. tuberculosis H37Rv, with the widely used, attenuated laboratory variant, H37Ra. This analysis, combined with gene complementation studies, has identified a mutation in the two-component system regulator PhoP that impacts on the secretion and T cell recognition of the 6-kD early-secreted antigenic target ESAT-6. In previous studies, both PhoP and the ESAT-6 secretion system ESX-1 have been identified independently as major virulence determinants of M. tuberculosis, so the finding that their action is interconnected opens exciting new insights and perspectives into the virulence regulation of tubercle bacilli.

## Introduction

125 years of intense research on the major human pathogen Mycobacterium tuberculosis have passed since its discovery by Robert Koch, resulting in a huge body of knowledge. In spite of the great progress that has been made in the understanding of some basic features of its pathogenesis, tuberculosis remains a major threat to human life in most parts of the world. Indeed, M. tuberculosis has retained many secrets of its so successful pathogenic lifecycle. Among the different possibilities to obtain new insight into the mechanisms employed by M. tuberculosis to infect its host, the analysis of attenuated strains is one promising approach, and there are some well-documented examples of laboratory-attenuated strains. One of them is the “Bacille de Calmette et Guérin” (BCG), which was originally derived in 1921 from a virulent Mycobacterium bovis strain by 230 passages on potato-glycerol-ox bile medium [1]. The genetic lesions of BCG have recently been determined [2–4], revealing that the loss of region of difference 1 (RD1), which encodes part of the ESX-1 secretion system [5], was one of the key events in its attenuation.

Another famous example of an attenuated strain is M. tuberculosis H37Ra (“a” for avirulent) (H37Ra). This strain was obtained in 1934 by serial passage of patient isolate M. tuberculosis H37 through media with different pHs [6] and since then has been widely used in many laboratories in the world. Despite its long use, the reasons for its stable attenuation have not yet been elucidated. As H37Ra is derived from the same parent strain as M. tuberculosis H37Rv (“v” for virulent) (H37Rv), the sequenced paradigm strain of tuberculosis research [7], genomic comparisons of the attenuated and virulent variants of M. tuberculosis H37 are particularly interesting and have the potential to identify subtle genetic changes that might be responsible for the phenotypic differences observed between the two strains. In a previous study we have tried to reveal these determinants [8], but the methods employed only identified large genetic polymorphisms, associated with IS*6110*, which were not found to be responsible for the attenuation of H37Ra [8]. In another study, Pascopella et al. [9] transformed a cosmid library of H37Rv into H37Ra and then selected for clones that were enriched on passage through the mouse. A number of overlapping cosmid clones that gave enhanced growth and survival in the spleens of infected mice relative to that of wild-type H37Ra were identified [9]. However, the effects of these complementation attempts on virulence remained limited, and no sequence information was described, which makes it difficult now to identify the genes implicated. H37Ra was also the subject of extensive micro-array based analyses, including whole genome comparative DNA/DNA analyses [10] and transcriptional studies [11,12], which have identified some candidate genes that were consistently downregulated. However, a definitive conclusion about the molecular determinants of the attenuation could not be drawn.

As all these previous attempts have failed to identify the genetic basis for the attenuation, we subjected H37Ra to microarray-based DNA re-sequencing (NimbleGen Systems). This technique has previously permitted single nucleotide polymorphisms (SNPs) of a PA-824 drug resistant mutant strain of H37Rv to be detected [13]. This approach was combined with gene “knock-in” strategies, to complement selected lesions, that allowed recombinant H37Ra strains to be engineered, whose virulence and immunogenicity were then evaluated in in vitro, ex vivo and animal models. This strategy led us to identify and characterize a point mutation in the *phoP*/*phoR* two-component regulatory system of H37Ra that has uncovered novel regulatory links, which impact on the secretion and T cell recognition of the major T cell antigens ESAT-6 and CFP-10.

## Results

### Microarray-Based Comparative Genome Sequencing of M. tuberculosis H37Ra

The genome-wide comparative mutational analysis of H37Ra and H37Rv was carried out by NimbleGen Systems following a previously published method [13]. Putative SNPs with high probability scores were separated into synonymous and non-synonymous SNPs of which the latter were verified by conventional dye terminator cycle sequencing. By this combined approach we identified 13 non-synonymous SNPs that differed between the H37Ra and H37Rv strains used (Table S1). We were particularly interested in a C to T mutation responsible for the serine to leucine replacement at position 219, S219L, of the two-component regulator PhoP as this protein is well known for its involvement in the virulent phenotype of M. tuberculosis [14]. Importantly, on inspection of the structure of the PhoP-ortholog from Bacillus subtilis, it was found that the equivalent residue Ser 207 is a main residue in the DNA-binding domain, helix alpha3 [15]. For the other 13 genes we found no indication that would identify them as potential virulence genes, as none of them belong to the 5% of genes that were previously determined by transposon site hybridization (TraSH) as being essential for in vivo growth of M. tuberculosis [16].

While writing this article, the whole genome sequence of H37Ra became publicly available (NC_009525), and comparison of the SNP data obtained from the H37Ra strain used in our laboratory (Table S1) with the NC_009525 data, showed that some differences existed between the H37Ra strains, a phenomenon which was also previously observed for BCG [4] and H37Rv [17]. Nevertheless, the S129L mutation in PhoP, which we consider an important SNP involved in the attenuation and reduced immunogenicity of H37Ra, is identical in all H37Ra strains.

### Rationale for Knock-Ins

To evaluate the phenotypic effect of the different SNPs and to assess their potential contribution to the attenuation process, we undertook functional genomic analyses using knock-ins of H37Ra, as described previously [18]. Clones spanning the different genomic regions of non-synonymous SNPs were selected from an ordered H37Rv library of integrating shuttle cosmids [7,19] and individually electroporated into H37Ra, where they inserted stably into the *attB* site. By this approach we obtained appropriately complemented transformants for the SNPs in genes *fadE5*, *rpsL*, and *phoP* (Table S1), using cosmids I230, I563, and I36, respectively. Based on the known role of *phoP* in virulence [14], the H37Ra strain complemented with I36 (H37Ra::*phoP*) was accorded highest priority for further molecular characterization and functional analyses, whereas the two other recombinants (H37Ra::*fadE5*, H37Ra::*rpsL*) served as controls. Figure S1 shows part of the nucleotide sequence of a PCR-amplified fragment obtained from H37Ra::*phoP*. It is clearly visible that at nucleotide position 656 of *phoP* two peaks exist, one originating from the SNP present in H37Ra and one from the integrated cosmid I36 carrying the H37Rv wild-type copy of *phoP*. Similar results were also obtained for the SNPs in *fadE5* and *rpsL* using cosmids I230 and I563, respectively (Figure S1). Correct integration of I36 was also confirmed by Southern blot. As depicted in Figure S2, hybridization of *SpeI-*digested genomic DNA with a ^32^P labeled *phoP* probe resulted in two bands of different sizes, one corresponding to the *SpeI* fragment harboring the H37Ra *phoP* gene (50 kb), and the other to the larger *SpeI* fragment created by integration of the I36 cosmid into the genome of H37Ra at the *attB* site. The successful integration of the cosmid was also reflected by a change in colony morphology that is shown in Figure S3. Indeed, Steenken and colleagues originally selected the H37Ra mutants mainly on the basis of the changes in colony morphology [6].

### Ex Vivo Virulence Studies

**Figure 1:**
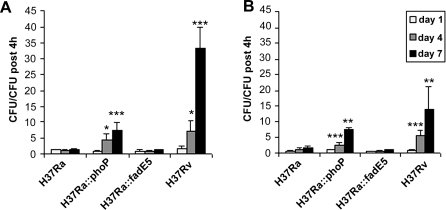
Macrophage Infection Studies Bone marrow–derived murine macrophages (BMM) were infected with M. tuberculosis H37Ra, H37Ra::*fadE5*, H37Ra::*phoP*, and H37Rv at a MOI of ca. 1:1 and 10:1 bacteria per cell (A and B, respectively). The figure shows the means and standard deviations of CFU ratio values (CFU at days 1, 4, and 7 relative to CFU at 4 h) obtained in a representative experiment performed in quadruplicate. The significant difference levels in growth characteristics between H37Ra and other strains (H37Ra::*phoP* and H37Rv) were determined by analysis of variance (ANOVA) (*p < 0.025, **p < 0.01, ***p < 0.005).

Changes in the regulatory potential of a pathogen are often accompanied by altered virulence. In a first attempt to determine the virulence of the complemented H37Ra knock-in strains relative to wild-type H37Ra and H37Rv, bone marrow–derived murine macrophages (BMM) were infected with the different strains at a multiplicity of infection (MOI) of 1:1 and 10:1 bacteria per cell. As depicted in Figure 1, macrophages were able to control ex vivo growth of H37Ra, but failed to control growth of H37Rv, confirming results from Freeman and colleagues [20]. When H37Ra knock-ins were tested, important differences in their ex vivo growth characteristics were found. Whereas H37Ra::*fadE5* (Figure 1) and H37Ra::*rpsL* (unpublished data) showed very little or no growth over the 7-d period, the H37Ra::*phoP* mutant grew more vigorously, with a 7.5-fold increase in colony-forming units (CFU) over the 7-d period. From these experiments we concluded that complementation of H37Ra with the *phoP* wild-type copy partially restored its virulence, but not to the extent of the fully virulent H37Rv strain.

### Virulence Studies of H37Ra Complemented Mutants in a Mouse Model

**Figure 2:**
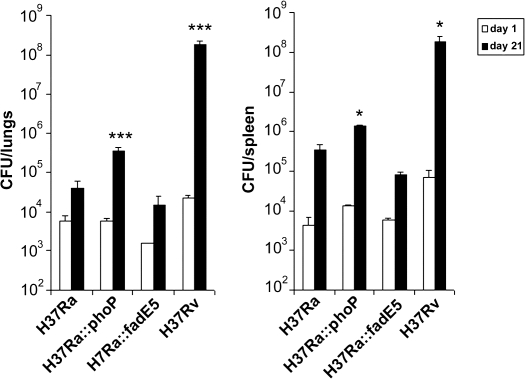
Virulence in the Murine Model Number of colony-forming units (CFU) in the lungs and spleens of severe combined immunodeficient (SCID) mice 1 d and 3 wk after intravenous (i.v.) infection with ca. 10^6^ CFU. The significance of differences between CFU values obtained in mice infected with H37Ra and other strains (H37Ra::*phoP* and H37Rv) was determined by ANOVA (*p < 0.025, ***p < 0.005).

Further assessment of the in vivo growth of different H37Ra knock-in strains was carried out by intravenous infection of severe combined immuno-deficient (SCID) mice. Complementation of H37Ra with the PhoP-expressing cosmid increased the virulence of the H37Ra::*phoP* recombinant relative to H37Ra, resulting in a 1.0 log and 0.5 log increase in CFU number in lungs and spleens, respectively. In contrast, no effects on the virulence were observed when H37Ra was complemented with *fadE5* (Figure 2) or *rpsL* (unpublished data). However, as already observed in macrophages, integration of *phoP* did not restore levels of virulence to those of the reference strain H37Rv (Figure 2). This situation is also reflected in the sizes of spleens, which correlate with the CFU data in spleens (Figure S4).

Together with the data from the macrophage infection assay, the results from the mouse infection show that the S219L mutation in the *phoP* gene definitely represents one genetic lesion that contributed to the attenuation of the H37Ra strain.

### Link between Mutation in *phoP* and Secretion of ESAT-6

**Figure 3:**
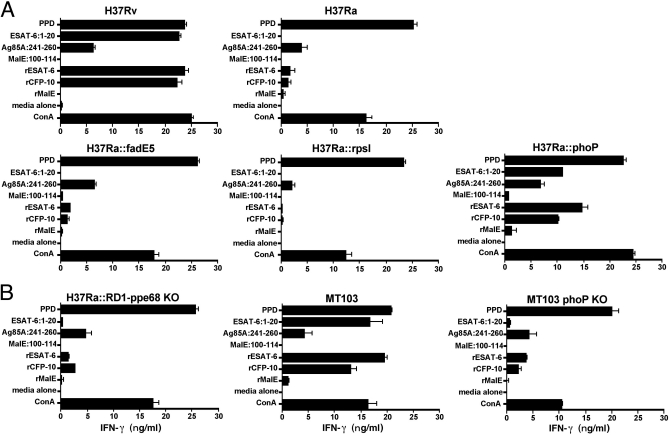
T Cell Responses in Mice ESAT-6- and CFP-10-specific T cell responses induced by various M. tuberculosis strains 2 wk after immunization. IFN-γ production by splenocytes in response to different antigens is expressed in ng/ml. (A) First panel of strains tested. (B) Second panel of strains tested for confirmation purposes.

As PhoP fulfills important regulatory functions in M. tuberculosis [21,22], it was of primary interest to identify and study potential effector molecules whose involvement in host pathogen interaction were influenced by the point mutation in *phoP* of H37Ra. Since secreted proteins of the ESX-1 system of M. tuberculosis constitute a major interface between the bacterium and its host [23], we analyzed the different strains for their potential to induce T cell responses against ESAT-6 and its binding partner, the 10-kD culture filtrate protein CFP-10. Groups of C57BL/6 mice were subcutaneously inoculated with H37Rv, H37Ra, or one of three recombinant H37Ra strains complemented with *phoP*, *fadE5*, or *rpsL*, respectively. Two weeks after vaccination we assessed the interferon-γ (IFN-γ) production of splenocytes mounted against ESX-1 antigens or controls. As anticipated, all tested strains induced IFN-γ production in response to purified protein derivative (PPD) but not to a control peptide (Mal-E), indicating successful vaccination (Figure 3).

Most importantly, the various strains differed extensively in their potential to induce antigen specific T cell responses towards ESAT-6 and CFP-10. As shown in Figure 3, splenocytes from mice that were inoculated with H37Rv produced high amounts of IFN-γ upon stimulation with ESAT-6 or CFP-10, whereas the responses of H37Ra, H37Ra::*fadE5*, and H37Ra::*rpsL* infected mice were extremely weak. In contrast, splenocytes from H37Ra::*phoP* inoculated mice showed very strong IFN-γ production in response to incubation with ESAT-6 and CFP-10.

**Figure 4:**
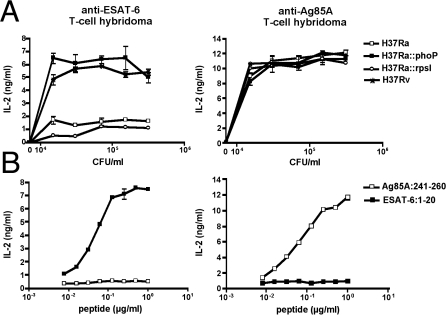
Antigen-Specific IL-2 Production of T Cell Hybridomas Analysis of IL-2 secretion by anti-ESAT-6 or anti-Ag85A T cell hybridomas in response to recognition of antigen presented by bone marrow–derived dendritic cells (BM-DC) incubated with homologous or control peptides or infected with H37Rv, H37Ra or recombinant H37Ra strains.

Exactly the same trend was observed when a highly sensitive T cell hybridoma assay was used to investigate ESAT-6 secretion. This test is based on the presentation of the immunodominant epitope contained in the first 20 amino acids of ESAT-6 by H37Ra, recombinant H37Ra, or H37Rv infected bone marrow–derived dendritic cells (BM-DC) to an ESAT-6-specific T cell hybridoma (NB11), restricted by I-A^b^. Figure 4A shows that infection of BM-DC with H37Ra or H37Ra::*rpsL* induced no stimulation of the hybridoma as judged by IL-2 production, while H37Ra::*phoP* or H37Rv triggered high amounts of IL-2 production in this very sensitive and specific test. All strains behaved comparably towards the Ag85A:241–260 control, emphasizing the specificity of the observed phenomenon for ESAT-6 (Figure 4B).

To further evaluate the involvement of *phoP* and the ESX-1 system in immunogenicity, C57BL/6 mice were vaccinated with additional strains. Firstly, we constructed a partially diploid H37Ra knock-in strain carrying cosmid RD1-*ppe68*-ko that, in BCG yields the greatest amounts of ESAT-6 expression and secretion [24]. However, in H37Ra the presence of this cosmid could not increase the weak ESAT-6 and CFP-10 T cell responses (Figure 3B) in the splenocyte IFN-γ assay. Secondly, we also tested the previously described M. tuberculosis MT103 *phoP* knock-out (ko) strain SO2 [14,25] in this assay and found that this strain induced a potent PPD response as previously reported [25]. However, in comparison to the corresponding MT103 wild-type strain, the SO2 strain showed a strongly reduced ESAT-6 and CFP-10 specific T cell response, corresponding to a similar low level as the H37Ra strain (Figure 3B).

Altogether, our data obtained from multiple replicate experiments indicate that a direct link exists between a fully functional PhoP and the ability to generate T cell responses against ESAT-6 and CFP-10.

### Functional Characterization of Knock-In Mutants

**Figure 5:**
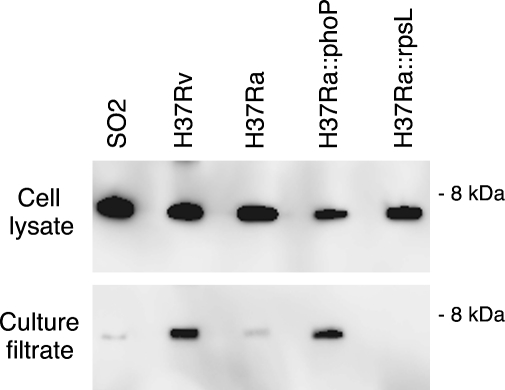
Secretion Analysis In vitro expression and secretion of ESAT-6 from M. tuberculosis H37Rv, H37Ra, MT103 *phoP* ko (SO2), and recombinant H37Ra complemented with integrating cosmids carrying genes *phoP* or *rpsL* from H37Rv. Total protein concentrations were determined by using Bio-Rad protein assay, and 15-μg samples were subjected to SDS-PAGE. Detection was carried out by using monoclonal anti-ESAT-6 antibody Hyb 76–8a.

We have previously shown that antigen-specific IFN-γ production of splenocytes is a reliable readout system to evaluate whether or not ESAT-6 was secreted by recombinant strains [23,24,26]. Thus, in order to determine the reason for the observed failure of H37Ra to induce ESAT-6-specific responses, H37Rv and H37Ra strains were tested for their potential to express and secrete ESAT-6 in vitro. The western blot analysis presented in Figure 5 shows that the cell lysates of H37Ra and H37Ra::*rpsL* contain large amounts of ESAT-6, indicating that the antigen is properly expressed. However, hardly any ESAT-6 was present in the culture filtrates of these cultures, indicating that these strains were unable to secrete ESAT-6 under the in vitro conditions employed in spite of proper expression. Similar results were obtained for H37Ra::*fadE5* (unpublished data). In contrast, from examination of the western blots of M. tuberculosis H37Ra::*phoP* and H37Rv it is clear that a large portion of their ESAT-6 protein is secreted into the culture filtrates.

Analysis of the M. tuberculosis MT103 *phoP* ko strain SO2 showed strong expression of ESAT-6, but only very little secreted ESAT-6 (Figure 5). In a previous report [25], the presence of ESAT-6 in cell free extracts was described for this strain, but no secretion assay was performed. Together, our findings correlate perfectly with the in vivo data described above and suggest that the lack of ESAT-6 specific T cell recognition for H37Ra, H37Ra::*fadE5*, H37Ra::*rpsL*, and SO2 is not caused by a loss of ESAT-6 expression, but rather due to a failure of (*phoP* dependent) ESAT-6 secretion.

The observation that knocking-in the ESX-1 region of H37Rv into H37Ra (H37Ra::RD1-*ppe68*-ko), and the resultant diploidy, did not improve the ESAT-6-specific T cell responses (Figure 3B), further argues that PhoP-dependent ESAT-secretion might be regulated via a mechanism that lies outside the RD1 region.

**Figure 6:**
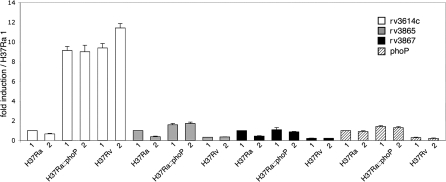
Quantitative RT-PCR Expression levels of genes *rv3614c*, *rv3865*, *rv3867*, and *phoP* in duplicate cultures of M. tuberculosis H37Ra and H37Ra::*phoP* and H37Rv evaluated by quantitative RT-PCR (qRT-PCR). Genes *rv3865* and *rv3867* were included as controls as these genes are encoded in the region of difference 1 (RD1) and show similarity to *rv3615/14c.* The expression level of each gene in each strain is reported as the ratio between the expression level in M. tuberculosis H37Ra culture 1, used as reference. For each strain, values were normalized to the level of 16S rRNA. Note that in M. tuberculosis a single *rrs* gene is present. Primer and probe sequences of genes *rv3614c*, *rv3865*, *rv3867*, *phoP*, as well as of 16S rRNA are listed in Table S2.

As closer inspection of the available literature on transcriptional analyses of a H37Rv-*phoP* ko strain [21] and H37Ra [12] suggested that the expression of gene cluster *rv3612c*-*rv3616c* is reduced in both strains, we evaluated the expression level of *rv3614c* in H37Rv, H37Ra, and H37Ra::*phoP* by quantitative real time PCR (qRT-PCR). In previous studies *rv3614c*, *rv3615c*, *rv3616c* (*espA*) were independently shown to be essential for proper ESAT-6 secretion [27,28]. We confirmed the trend that *rv3614c* was expressed much lower in H37Ra compared to H37Rv by qRT-PCR (Figure 6), whereas expression of *rv3614c* in H37Ra::*phoP* was restored to wild-type levels, suggesting that the *rv3612c*-*rv3616c* gene cluster is regulated by PhoP. Interestingly, expression of *phoP* was higher in H37Ra than in H37Rv (Figure 6). These findings are consistent with data from a previous transcriptional study [12] and suggest that the proposed ability of PhoP to downregulate its own expression [22] is affected by the S219L *phoP* mutation [29]. They also indicate that it is not a lack of *phoP* expression that is causing the *phoP*-associated effects in H37Ra.

## Discussion

The attenuated H37Ra strain was obtained at the Trudeau Institute in the 1930s in an attempt to dissociate virulent and avirulent forms of the tubercle bacillus H37. Steenken et al. have shown that the virulence of the H37 strain was associated with colony morphology and that the avirulent variant H37Ra failed to propagate in an ordinarily susceptible host while the virulent variant H37Rv was able to proliferate [6]. Although these different H37 daughter strains have been available for many years now and belong to the standard strain collections of almost every tuberculosis diagnostic or research laboratory in the world [30,31], the genetic basis of the decreased virulence of H37Ra remained unknown.

The identification of the amino acid change S219L in the predicted DNA binding region of the C-terminal effector domain of PhoP [15,22] in H37Ra was therefore a particularly exciting finding that has the potential to explain part of the attenuation of the H37Ra strain and to elucidate the PhoP/PhoR associated complex regulatory network in M. tuberculosis. In the bacterial world the PhoP/PhoR like two-component systems are widely distributed and fulfill a large variety of functions including sensing phosphate and magnesium [32,33], or regulating virulence genes [34]. For M. tuberculosis, several studies have described a link between the inactivation of *phoP* and loss of virulence, suggesting that the *phoP* gene is required for intracellular growth of M. tuberculosis [14,25]. It was also found that the PhoP-PhoR system controls the biosynthesis of polyketide-derived lipids [35] and determines the amount of triacylated and monoacylated lipoarabinomannans in M. tuberculosis [36]. However, according to very recent information, these polyketide-derived (Pks2–4) lipids do not have a strong impact on the virulence of M. tuberculosis [29]. Finally, microarray-based trancriptome analysis using the wild-type versus a *phoP* ko mutant of H37Rv has provided an overview of the transcriptional regulation linked to PhoP [21]. That study identified 44 genes in the *phoP*-ko strain that were expressed at least 2.5-fold lower in the mutant than their counterparts in the M. tuberculosis wild-type strain.

Most interestingly, about two thirds of these 44 genes were also found substantially lower expressed in H37Ra in comparison to H37Rv, when the transcriptional profiles of H37Ra and H37Rv cultures grown in various media were examined [12]. This finding argues that the PhoP S219L mutation likely has a broad impact on gene regulation in H37Ra with effects similar to those seen for complete inactivation of *phoP*. Among the genes expressed at low level in the *phoP*-ko strain and in H37Ra were several that are involved in lipid metabolism; for example, the polyketide synthases *pks2* (*rv3825c*) or the Pks-associated protein *papA1* (*rv3824*c). There were also several *ppe* genes that seem to be regulated by *phoP* but not *ppe68* [21]. In addition, NirA (Rv2391), a putative nitrite reductase and MmpL10-FadD21 (Rv1183-85c), which were all identified by TraSH analysis as potentially implicated in virulence [16], seem to be regulated by PhoP [12,21].

However, the most striking link in relation to our results on *phoP* and ESAT-6 secretion that was revealed by these two transcriptional analyses, were the data on gene cluster *rv3612c-rv3616c*. Closer inspection of the data published in the corresponding supplemental materials showed that *rv3612c*-*rv3616c* were strongly downregulated in the H37Rv *phoP* ko mutant as well as in H37Ra [12,21]. These transcriptome data corroborate our results obtained by quantitative RT-PCR using probe rv3614c, which showed that gene *rv3614c* was definitely expressed much lower in H37Ra than in H37Rv and H37Ra::*phoP* strains (Figure 6). Since in previous, independent studies it has been demonstrated that functions of the *rv3614c-rv3616c* (*espA*) gene cluster were essential for the secretion of ESAT-6 and CFP-10 [27,28], it is quite probable that the lack of ESAT-6-specific T cell responses observed for strains H37Ra and SO2 (Figure 3) might be caused by the insufficient expression of *rv3612c-rv3616c*, which results from the lack of a fully functional PhoP in these strains. Moreover, the expression values of genes *rv3612c-rv3616c* in H37Rv described in the article by Gao et al. [12] show substantial differences between cultures grown in roller bottles relative to cultures grown in shake flasks, arguing that this gene cluster might be regulated by environmental conditions through the PhoP/PhoR system. It is not clear yet which factors might contribute to this effect. However, upregulation of *phoP* and *rv3614c-rv3616c* was observed after incubation of M. tuberculosis in 5mM H_2_O_2_ [37]. Furthermore, *rv3614c-rv3616c* were reported to be transiently upregulated in the phagosome and under acidic stress [38]. Altogether, from these reports it seems plausible that the pleiotropic regulator PhoP is involved in the regulation of *rv3612c-rv3616c* expression and thereby influences ESAT-6 secretion and virulence [16], but to unambiguously answer this question, virulence tests with recombinant H37Ra and/or *phoP* ko strains that express *rv3616c-3612c* under a constitutive promoter or a tetracycline inducable expression system [39] will be needed. Construction of such strains has recently been initiated as a first step of a future study to gain deeper insight into signaling events and regulation of the ESX-1 secretion system in different tubercle bacilli, including members of the putative progenitor species Mycobacterium prototuberculosis [40].

As in previous DNA/DNA microarray analyses [10], and from inspection of the very recently released genome sequence of H37Ra, no significant differences in the ESX-1 encoding region were found, the assumption that the observed lack of ESAT-6 secretion in H37Ra may be caused by factors outside of the RD1 region is very plausible.

In contrast, in another transcriptome analysis, Mostowy and colleagues [11] found several genes of the RD1 region, including that encoding ESAT-6, among the genes that were at least 2.5-fold lower expressed in H37Ra than in H37Rv. Our results obtained for ESAT-6 expression (Figure 5) and the transcriptome data from Gao and colleagues [12] argue against direct downregulation of ESAT-6 in H37Ra. However, it is also possible that the observed differences may be caused by variations of in vitro culture conditions.

In conclusion, in this work we report the identification and profound consequences of the PhoP S219L mutation on the widely used H37Ra strain. BLASTP comparisons of the PhoP sequence showed that the predicted DNA binding region, where the mutation occurred, is perfectly conserved among a wide range of actinobacteria, with H37Ra being the only strain showing such a mutation. By complementation with the wild-type copy of PhoP, we obtained a significant increase in virulence in ex vivo and animal models as well as restoration of ESAT-6 secretion and the accompanying antigen-specific immunological recognition by sensitized splenocytes or T cell hybridomas.

It should be mentioned here that the SO2 *phoP* ko strain, which showed strong ESAT-6 expression but little ESAT-6 secretion (Figure 5), induced only very reduced ESAT-6- specific T cell responses (Figure 3). This strain was previously used to vaccinate mice and guinea pigs and conferred good protection against a challenge with virulent M. tuberculosis [25]. As ESAT-6-specific T cell responses induced by this strain are low, ESAT-6-based IFN-γ production assays might be used to differentiate between vaccination with SO2 and infection with wild-type M. tuberculosis.

With its well documented role in virulence and strong T cell antigenicity, ESAT-6 is one of the most important proteins of M. tuberculosis involved in host pathogen interaction [5,41–43]. However, as previously observed in complementation studies of BCG [18], the level of virulence displayed by the *phoP* complemented H37Ra strain was still substantially below that of wild-type H37Rv, arguing that the strong attenuation of the H37Ra strain is likely based on several different genetic lesions, such as the one responsible for the absence of phthiocerol dimycocerosates (PDIM) [29], that were probably introduced into this strain during years of continued culture following the original event of attenuation described by Steenken et al. in 1934 [6]. Indeed, preliminary comparison of the 4.4-Mb genome of H37Rv [7] (NC_000962) with the recently accessible H37Ra genome (NC_009525) revealed 194 differences, including 126 SNPs, 16 deletions, 27 insertions, and 25 substitutions. At present, we do not know how many of these differences actually contribute to the attenuation of H37Ra, and it also remains to be determined how many differences exist among the various H37Ra strains that are presently used in the worldwide tuberculosis research laboratories. The link described here between mutation of *phoP* and consequences on ESAT-6 secretion, however, appears to be one factor that significantly contributes to attenuation.

Finally, it is intriguing that both BCG and H37Ra, the two most often used attenuated tubercle bacilli, are attenuated for reasons linked to loss of ESAT-6 functions. Thus, our results once more emphasize the great importance of the ESX-1 system for tubercle bacilli and provide important new information about the *phoP*-associated virulence regulation in M. tuberculosis.

## Materials and Methods

### Bacterial strains and comparative genome sequencing.

M. tuberculosis H37Ra corresponding to ATCC 25177 and M. tuberculosis H37Rv were taken from stocks held at the Pasteur Institute. H37Ra and H37Rv were grown in 400 ml of Dubos liquid medium (Difco) supplemented with albumin-dextrose-catalase (ADC, Difco) at 37 °C for 1 wk. Genomic DNA was made by standard methods [7].

Comparative mutational analysis of H37Ra and H37Rv was carried out by NimbleGen Systems following a previously published method using microarray-based sequencing arrays [13]. Verification of the indicated SNPs was done by dye terminator cycle sequencing of PCR-amplified H37Ra fragments following previously published methods [44].

For construction of H37Ra *fadE5*, *rpsL*, and *phoP* knock-ins, high concentrated plasmid DNA preparations from clones I230, I563, and I36, selected from an ordered M. tuberculosis H37Rv library of pYUB412 integrating shuttle cosmids [7,19], were individually electroporated into H37Ra, following a previously published protocol [45]. Transformants were selected on Middlebrook 7H11 medium (Difco) supplemented with oleic acid-albumin-dextrose-catalase (OADC, Difco) and 50 μg ml^−1^ hygromycin. Resistant colonies appearing after 4 wk were analyzed for the presence of the integrated genes *phoP*, *rpsL*, or *fadE5* by specific amplification and sequencing (Figure S1). Proper integration of the cosmid was further determined by Southern hybridization (Figure S2) following a previously published method [4]. The previously described M. tuberculosis MT103 *phoP* ko mutant SO2 [14,25], which carries an inactivated *phoP* gene due to the insertion of a kanamycin cassette, was used for control purposes in ESAT-6 expression and secretion assays.

### Macrophage virulence assays.

BMM were obtained as described [24] by seeding bone marrow cells from 8-wk-old C57BL/6 mice in a 96-well plate at a density of 4 × 10^4^ cells per well. Cells were cultured in RPMI medium supplemented with 10% heat-inactivated fetal calf serum, 10% L-cell-conditioned medium, and 2 mM L-glutamine. Culture medium was changed at day 4 and then every 3 d, with cells being differentiated into macrophages after 7 d. For the macrophage virulence assay, cells were infected with bacterial suspensions of H37Rv, H37Ra, and H37Ra knock-ins at a MOI of 1:1 and 10:1 (bacteria:cell). At 4 h, 1, 4, and 7 d after infection, macrophages were lysed by addition of 0.1% Triton X-100 (Fluka) in PBS and the number of intracellular bacteria was determined by plating serial dilutions of cell lysates on Middlebrook 7H11 medium supplemented with OADC.

### Virulence studies in mice.

Mycobacterial strains were grown and suspensions prepared as described previously [23]. 6-wk-old male C.B-17/Icr Ico SCID mice (Charles River) were infected intravenously with 10^6^ CFU. At 3 or 4 wk after infection mice were sacrificed and spleens and lungs homogenized using an MM300 apparatus (Qiagen) and 2.5-mm diameter glass beads. Serial 5-fold dilutions of organ homogenates were plated on Middlebrook 7H11 agar supplemented with 50 μg of hygromycin or ampicilin / ml^−1^ when appropriate, and the CFUs were counted after 3–4 wk of incubation at 37 °C.

### Splenocyte T cell response assays.

For splenocyte T cell response assays, adult C57BL/6 (H-2b) (Janvier) mice were injected subcutaneously with 10^6^ CFU of H37Rv, H37Ra, or recombinant H37Ra knock-in strains. 2 wk after immunization, splenocytes were cultured (10^6^ cells/well) with 10 μg/ml of purified protein derivatives (PPD) (Statens Serum Institute), 4 μg/ml of recombinant ESAT-6, CFP-10 (kindly provided by Marcella Simsova and Peter Sebo), or MalE as negative control, or with 10 μg/ml of synthetic peptides. After 72 h of incubation, amounts of IFN-γ were quantified in culture supernatants using a sandwich ELISA (detection limit of 500 pg/ml) with AN-18 and biotin-conjugated R4-6A2 monoclonal antibodies as described elsewhere [23]. Results are expressed as the mean and standard deviation of duplicate culture wells.

### Generation of anti-ESAT-6 T cell hybridomas and detection of ESAT-6:1–20 presentation upon infection of dendritic cells with mycobacteria.

T cell hybridomas specific for ESAT-6:1–20 peptide were generated from C57BL/6 (H-2^b^) mice immunized s.c. with 50 μg/mouse of the synthetic ESAT-6:1–20 peptide in Freund's incomplete adjuvant. 2 wk after immunization, total splenocytes were stimulated with 10 μg/ml of ESAT-6:1–20 peptide. At day 4, viable cells were harvested on Lympholyte M (Cedarlane Laboratories) and were fused with an equal number of BW51–47 thymoma cells by use of polyethylene glycol 1500 (Roche Diagnostics GmbH), as previously described [46]. Hybridomas were screened for their capacity to release IL-2 upon recognition of an immunodominant epitope contained in the ESAT-6:1–20 peptide. Briefly, bone marrow–derived dendritic cells (BM-DC) were stimulated overnight, in antibiotic-free conditions, with ESAT-6:1–20 peptide (1 μg/ml), recombinant ESAT-6 (1 μg/ml), or an ESAT-6-expressing BCG::RD1 [18], at a MOI of 1:1. BM-DC were then washed three times and co-cultured overnight with T cell hybridomas. The presence of IL-2 in the supernatant of co-cultures was monitored by standard IL-2-specific ELISA. L fibroblasts, transfected with I-A^b^ or I-A^d^ molecules, were used as peptide presenting cell, in the same type of assay for determination of the H-2 restriction of the antigen presentation to these T cell hybridomas. A selected T cell hybridoma (NB11), restricted by I-A^b^, with the detection limit of 6 nM of ESAT-6:1–20 peptide, was chosen to determine the capacity of H37Ra, H37Ra knock-ins, and H37Rv to induce presentation of ESAT-6:1–20 via the MHC-II pathway. An anti-Ag85A:241–260 T cell hybridoma, (DE10) [46], was used in parallel to detect the presentation of Ag85A:241–260 by I-A^b^.

### Preparation of cell lysates, supernatants, and western blotting.

Protein preparation of whole cell lysates and culture supernatants of mycobacterial cultures was done as previously described [24]. Total protein concentrations were determined by using a Bio-Rad protein assay (Bio-Rad), and 15-μg samples were subjected to sodium dodecyl sulfate-polyacrylamide gel electrophoresis (SDS-PAGE) using NuPage Bis-Tris Mini gels (Invitrogen) in NuPage MES running buffer following the manufacturer's recommendations. The immunoblot analysis was carried out with mouse ESAT-6 monoclonal antibody (Hyb 76–8; Antibody Shop, Statens Serum Institute) as described previously [24], after proteins were transferred onto nitrocellulose membranes using the iBlot Dry Blotting System (Invitrogen; Voltage 25V; Run time: 7 min).

### Reverse transcription.

To ensure the same amount of cDNA was analysed in every specimen, reverse transcription was performed as a separate step from PCR. The mRNA was then reverse transcribed into cDNA (Reverse-iT Max 1st Strand Synthesis Kit, Abgene). Briefly, 1 μg of RNA was added to a final reaction volume of 20 μl composed of 1 μl of random, 4 μl 10 × RT Buffer, 2 μl dNTP mix, 1 μl QRTase Enhancer, 1 μl reverse transcriptase and water. The reaction was vortexed briefly and incubated at 42 °C for 1 h. The tubes were then heated at 75 °C for 10 min to inactivate the reverse transcriptase. The cDNA samples were then purified using Qiaquick Columns (Qiagen, Incorporated) to remove unused dNTPs and any other DNA or RNA material <25 bases in length. The cDNA was stored at −20 °C prior to analysis by real-time PCR.

### Quantitative real-time PCR.

Primers and probes used in qRT-PCR are listed in Table S2. To perform qRT-PCR, 5 dilutions of M. tuberculosis H37Rv cDNA were used as PCR templates for each standard curve. Each standard dilution and each sample was tested in duplicate and negative controls were included in each experiment. Each amplification was performed in a 96-well PCR plate covered with optical tape in the AB7300 real-time PCR instrument (Applied Biosystems). A final volume of 20 μl was used containing 5 ng of cDNA template, 10 μl of TaqMan Master Mix (Applied Biosystems), 1 μl Mix containing Primers and Probes, and water. The reaction was subjected to denaturation at 95 °C for 2 min followed by 40 cycles of denaturation at 95 °C for 45 s, annealing/elongation at 60 °C for 1 min. Fluorescent data were specified for collection at the end of the annealing/elongation step in each cycle.

For each gene, a standard curve of amplification was performed using known amount of cDNA from H37Rv. Based on the CT values obtained, a linear regression line was plotted using a custom made software supplied with the AB7300 real-time PCR instrument and the resulting equation is used to calculate the efficiency of amplification. To normalize the results between samples, the 16S rRNA gene and the H37Ra strain (culture number 1) were used as reference gene and reference strain, respectively. The expression level of each gene was reported as ratio of expression (fold induction) compared to the expression level in H37Ra (culture 1), using following calculation: ratio of induction = Efficiency of amplification _(gene of interest)_^−ΔΔCT^_,_ where ΔCT = CT_(gene of interest)_ − CT_(16S rRNA)_ in each strain tested, and ΔΔCT = ΔCT_(strain tested)_ − ΔCT _(H37Ra 1)_.

## Supporting Information

Figure S1 Partial Sequences of Genes *rpsL*, *fadE5*, and *phoP* in M. tuberculosis H37Ra and H37Ra Knock-In Strains Arrows indicate the nucleotide variation at the corresponding SNP site resulting from complementation with the wild-type gene from M. tuberculosis H37Rv.
(212 KB PDF)

Figure S2 Restriction and Hybridization Profiles of M. tuberculosis H37Ra and H37Ra::*phoP*
*SpeI*-digested, pulsed field gel electrophoresis (PFGE) profiles of M. tuberculosis H37Ra and H37Ra::*phoP* (left panel), and after hybridization with a radiolabeled phoP probe (right panel).
(155 KB PDF)

Figure S3 Colony Morphology of Different M. tuberculosis H37 Strains (35 KB PDF)

Figure S4 Sizes of Spleens from SCID Mice SCID mice were intravenously infected with different M. tuberculosis H37 strains (10^6^ bacteria) for 3 wk (A) or 4 wk (B).
(59 KB PDF)

Table S1 Identity and Genomic Position of Mutations in the M. tuberculosis H37Ra Strain Used in This Study (48 KB DOC)

Table S2 Primers and Probes Used for Quantitative RT-PCR (22 KB DOC)
