# Supplementary material for: Control of M. tuberculosis ESAT-6 Secretion and Specific T Cell Recognition by PhoP
Source: PLoS Pathog. 2008 Feb 15;4(2):e33. doi: 10.1371/journal.ppat.0040033 (PMC2242835; doi:10.1371/journal.ppat.0040033)
Supplement: Supplementary file 4 [file ppat.0040033.sg004.pdf]

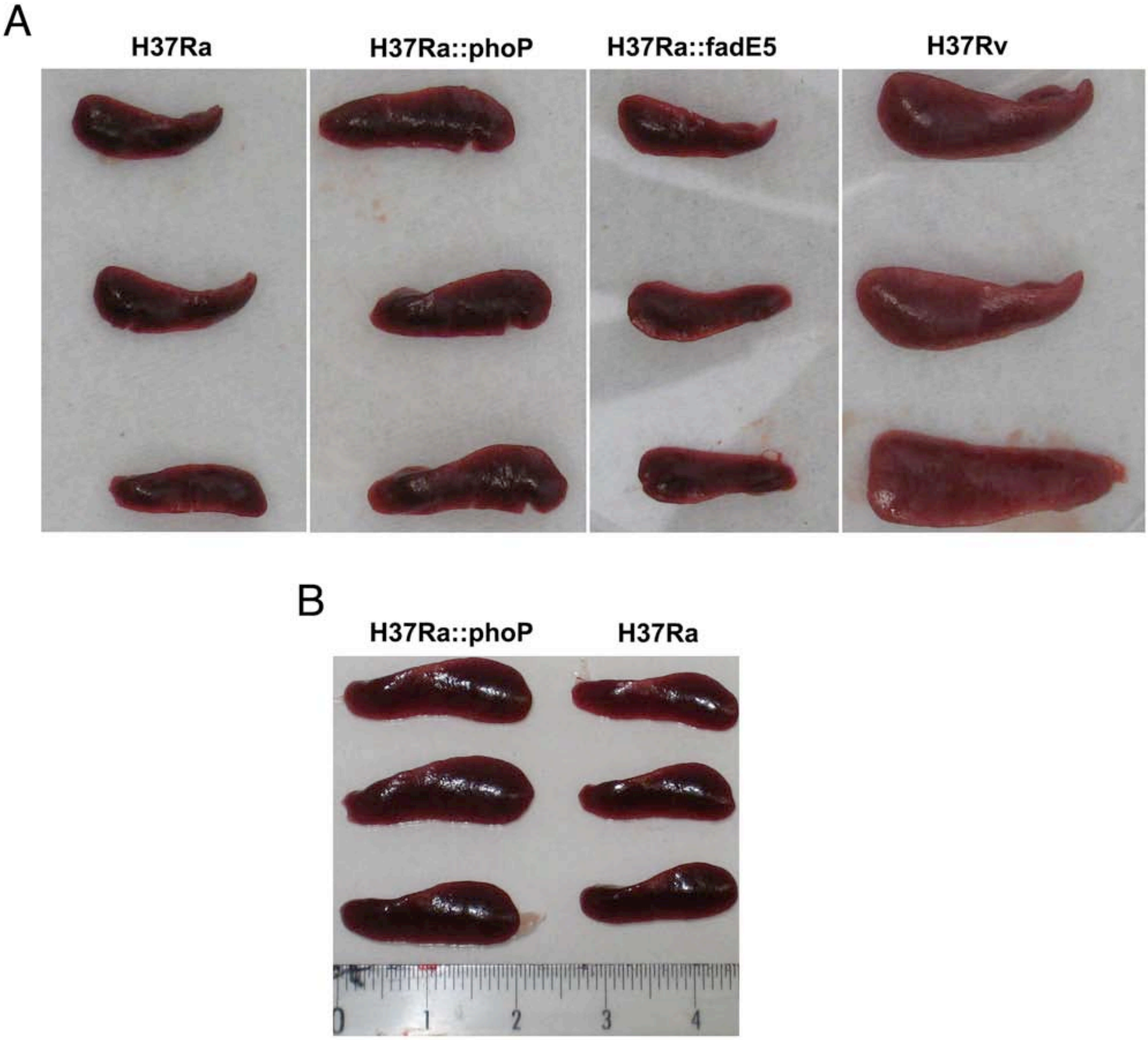
A
H37Ra
H37Ra::phoP
H37Ra::fadE5
H37Rv
B
H37Ra::phoP
H37Ra
0
1
2
3
4
