# Supplementary material for: Control of M. tuberculosis ESAT-6 Secretion and Specific T Cell Recognition by PhoP
Source: PLoS Pathog. 2008 Feb 15;4(2):e33. doi: 10.1371/journal.ppat.0040033 (PMC2242835; doi:10.1371/journal.ppat.0040033)
Supplement: Supplementary file 5 [file ppat.0040033.st001.doc]

**Table S1. Identity and Genomic Position of Mutations in the *M. tuberculosis* H37Ra Strain Used in This Study**

| Gene name | Rv-name | position (H37Rv) | SNP-type | position mutation | Verification SNP | Amino-Acid changes |
| --- | --- | --- | --- | --- | --- | --- |
|  |  |  |  |  |  |  |
| *rv0064* | Rv0064 | 68620-71556 | G-A | 69989 | OK | G-D |
| *nrp* | Rv0101 | 110001-117536 | T-G | 116000 |  | V-V |
| ***fadE5*** | **Rv0244c** | **293801-295633** | **G-T** | **294367** | **OK** | **G-C** |
| *rv0323* | Rv0323 | 390583-391251 | T-C | 390828 | OK | S-G |
| *rv0397* | Rv0397 | 475816-476181 | C-G | 475866 |  | T-T |
| *rv0504* | Rv0504 | 594805-595302 | A-C | 595039 | ND | F-L |
| *hemC* | Rv0510 | 601857-602783 | A-C | 602263 | OK | E-A |
| *mmaA2* | Rv0644c | 738300-739160 | A-G | 739119 |  | A-A |
| ***rpsL*** | **Rv0682** | **781560-781931** | **C-T** | **781815** | **OK** | **R-W** |
| ***phoP*** | Rv757 | **851608-852348** | **C-T** | **852263** | **OK** | **S-L** |
| *rv0919* | Rv0919 | 1024684-1025181 | T-C | 1025106 |  | F-F |
| *rv0966* | Rv0966 | 1077236-1077835 | A-G | 1077312 | OK | V-A |
| *pks3/4* | Rv1180 | 1313725-1315188 | A-C | 1315191 | OK | *-Y |
| *pks4* | Rv1181 | 1315234-1319979 | G-A | 1315884 |  | A-A |
| rv1194 | Rv1194 | 1337251-1338513 | C-T | 1338240 | OK | V-M |
| *pknH* | Rv1266 | 1413963-1415840 | C-T | 1414021 | OK | R-Q |
| *ppe33* | Rv1809 | 2051282-2052685 | T-C | 2051746 |  | A-A |
| *rv1815* | Rv1815 | 205758-2058190 | A-T | 2057774 | OK | I-F |
| *rv2037* | Rv2037 | 2282750-2283721 | C-T | 2282787 | OK | C-Y |
| *pdhC* | Rv2495 | 2808761-2809939 | T-C | 2809621 | OK | T-A |

Genes shown in bold were succesfully complemented with integrating constructs and their phenotypes further determined. Gray background = synonymous SNPs that were suggested by the resequencing approach, but which were not further verified. OK = done, ND = not determined.
