# Supplementary material for: Control of M. tuberculosis ESAT-6 Secretion and Specific T Cell Recognition by PhoP
Source: PLoS Pathog. 2008 Feb 15;4(2):e33. doi: 10.1371/journal.ppat.0040033 (PMC2242835; doi:10.1371/journal.ppat.0040033)
Supplement: Supplementary file 6 [file ppat.0040033.st002.doc]

**Table S2. Primers and Probes Used for Quantitative RT-PCR**

| Target Gene | Primers | Probes |
| --- | --- | --- |
| *rv3614c* | GCACGGTGTCGGTATCGA | 6FAM-ACGGCCGGATTGACCA |
|  | TGGCCGACAGCTCGAC |  |
| *rv3865* | CGAGTTCAGCTTACCCATGGTT | 6FAM-TTCACGTCGAAATTC |
|  | CAAACTCTTGCAGCGTGTCATT |  |
| *rv3867* | GCTGGCCGACGAGATCTT | 6FAM-CAGATCGGCAATAACG |
|  | GCCCGCGCCTTTTGG |  |
| *phoP* | ACTATGTGACAAAGCCCTTCAGTTT | 6FAM-CTGGCCACGACCTCC |
|  | CGCGTCGCAGGATGAC |  |
| 16S-RNA gene | GTGGACTACCAGGGTATCTAATCCT | 6FAM-TCCCCACGCTTTCG |
|  | GGGTCTCTGGGCAGTAACTG |  |

Primers and probes were designed by custom TaqMan MGB gene expression assays service (Applied Biosystems, Foster City, USA).
